# The primary function of Six5 of *Fusarium oxysporum* is to facilitate Avr2 activity by together manipulating the size exclusion limit of plasmodesmata

**DOI:** 10.3389/fpls.2022.910594

**Published:** 2022-07-29

**Authors:** Mila C. Blekemolen, Lingxue Cao, Nico Tintor, Tamara de Groot, Diana Papp, Christine Faulkner, Frank L. W. Takken

**Affiliations:** ^1^Molecular Plant Pathology, Swammerdam Institute of Life Science (SILS), University of Amsterdam, Amsterdam, Netherlands; ^2^The John Innes Centre, Norwich Research Park, Norwich, United Kingdom

**Keywords:** plant- fungal interactions, effectors, *Fusarium oxysporum*, plasmodesmata, cell-to-cell movement, defense responses, virulence

## Abstract

Pathogens produce effector proteins to manipulate their hosts. While most effectors act autonomously, some fungal effectors act in pairs and rely on each other for function. During the colonization of the plant vasculature, the root-infecting fungus *Fusarium oxysporum (Fo)* produces 14 so-called Secreted in Xylem (SIX) effectors. Two of these effector genes, *Avr2 (Six3)* and *Six5*, form a gene pair on the pathogenicity chromosome of the tomato-infecting *Fo* strain. Avr2 has been shown to suppress plant defense responses and is required for full pathogenicity. Although Six5 and Avr2 together manipulate the size exclusion limit of plasmodesmata to facilitate cell-to-cell movement of Avr2, it is unclear whether Six5 has additional functions as well. To investigate the role of Six5, we generated transgenic Arabidopsis lines expressing *Six5*. Notably, increased susceptibility during the early stages of infection was observed in these *Six5* lines, but only to *Fo* strains expressing *Avr2* and not to wild-type Arabidopsis-infecting *Fo* strains lacking this effector gene. Furthermore, neither PAMP-triggered defense responses, such as ROS accumulation and callose deposition upon treatment with Flg22, necrosis and ethylene-inducing peptide 1-like protein (NLP), or chitosan, nor susceptibility to other plant pathogens, such as the bacterium *Pseudomonas syringae* or the fungus *Verticilium dahlia*, were affected by *Six5* expression. Further investigation of the ability of the Avr2/Six5 effector pair to manipulate plasmodesmata (PD) revealed that it not only permits cell-to-cell movement of Avr2, but also facilitates the movement of two additional effectors, Six6 and Six8. Moreover, although Avr2/Six5 expands the size exclusion limit of plasmodesmata (i.e., gating) to permit the movement of a 2xFP fusion protein (53 kDa), a larger variant, 3xFP protein (80 kDa), did not move to the neighboring cells. The PD manipulation mechanism employed by Avr2/Six5 did not involve alteration of callose homeostasis in these structures. In conclusion, the primary function of Six5 appears to function together with Avr2 to increase the size exclusion limit of plasmodesmata by an unknown mechanism to facilitate cell-to-cell movement of *Fo* effectors.

## Introduction

Pathogens can cause severe damage to plants, resulting in devastating crop losses in many agronomically important crops worldwide (Fisher et al., 2012). Plant pathogens (including bacteria, fungi, and oomycetes) employ small, secreted proteins, called effectors, to manipulate their hosts (Lo Presti et al., 2015; Toruño et al., 2016). An increasing number of cases have been reported where plant pathogens manipulate plasmodesmata (PD) to promote pathogenesis. PD channels connect neighboring cells, traversing the plant cell wall, and thereby act as direct connections between plant cells for communication and movement of small proteins, metabolites, and ions. PD in different developing cells and tissues vary in their transport capabilities. A tightly regulated PD function is essential to allow the controlled distribution of developmentally important molecules and thereby for normal plant development. The number of PD in root tissue, often an entry point for plant pathogens, increases from the epidermis toward the vascular tissues (Burch-Smith et al., 2011). Manipulation of PD channels can among others, benefit the spread of viral particles, fungal hyphae, or translocation of effector proteins to neighboring cells to potentially suppress immune responses (Benitez-Alfonso et al., 2010; Cheval and Faulkner, 2018; Liu et al., 2021). Although PD manipulation by viruses has been studied extensively, how filamentous pathogens manipulate PD with the aid of intracellular effectors is a relatively new and unexplored field (Liu et al., 2021). Effector proteins PWL2 and BAS1 of the rice blast fungus *Magnaporthe oryzae* spread into uninfected cells through PD when delivered into the cytoplasm of rice cells via a biotrophic interfacial complex (BIC) (Khang et al., 2010). Likewise, the maize smut fungus *Ustilago maydis* Cmu1 effector is also likely to spread to neighboring cells through PD (Djamei et al., 2011). Currently, it is unknown whether the PD-mediated translocation in these cases is a passive or an active process that is directed by the action of (additional) effector proteins. The *Phytophthora brassicae* effector RxLR3 inhibits callose synthases (CalS1, CalS2, and CalS3) at PD, thereby reducing the formation of callose deposits, which in turn increased the size exclusion limit (SEL) of PD to permit cell-to-cell movement of larger cargo (Tomczynska et al., 2020). Likewise, the Avr2(Six3)/Six5 effector pair of *Fo* was reported to interact at PD to facilitate the translocation of Avr2 to neighboring cells (Cao et al., 2018). PD-related genes (e.g., *LYK4, LYM2*, and *PDLPs*) are differentially regulated upon Fusarium infection of Arabidopsis, implying that PD manipulation during Fo infection is common for different plant species (Guo et al., 2021). Unanswered questions are how fungal effectors manipulate PD, to what extent the SEL is altered, and whether interference with callose deposition is a common theme. The Avr2/Six5 pair forms an excellent model to study this process in more detail due to the well-characterized functions of Avr2 (Di et al., 2017) and the possibility to test the effectors alone and in combination separating their functions on PD and other host processes.

*Fusarium oxysporum* is a root-infecting pathogen that causes vascular wilt disease in a wide variety of plants (Michielse and Rep, 2009). The host range of a single strain is typically restricted to one or a few plant species, e.g., *F. oxysporum* f.sp. *lycopersici* (*Fol*) infects tomatoes while *Fo5167* is pathogenic to Arabidopsis. *Fol* is able to direct its growth toward tomato roots by sensing peroxidases secreted in the tomato root exudates (Nordzieke et al., 2019). *Fo* invades the root system via small wounds or cracks in the epidermis that are often caused by lateral root formation. In Arabidopsis, the fungal hyphae can directly enter via the root tip (de Lamo and Takken, 2020; Redkar et al., 2022b). Fungal hyphae spread through the endodermis and cortical cells via the apoplast to eventually colonize the vasculature (de Lamo and Takken, 2020). During the infection process, effector proteins are secreted into the apoplastic spaces of the root cortex and into the xylem sap; fourteen of these *Fol*-secreted proteins have been identified as Six (Secreted In Xylem) proteins (Houterman et al., 2007; Schmidt et al., 2013; Redkar et al., 2022a). Some effectors function inside the apoplast, while others can be taken up by the host cell and exert their function intercellularly (e.g., Avr2, Six6, Six8) (Gawehns et al., 2014; Tintor et al., 2020). Six1, Avr2, Six5, and Six6 are required for full *Fol* pathogenicity, defining them as genuine effectors (Rep et al., 2004; Houterman et al., 2009; Gawehns et al., 2014; Ma et al., 2015). Avr2 suppresses PAMP-triggered immunity (PTI), a generic defense response triggered by the recognition of microbe-derived molecules or pathogen-associated molecular patterns (PAMPs) by cell surface-localized pattern-recognition receptors (PRRs). Well-studied examples of PAMP/PRR pairs are bacterial flagellin (and its immunogenic derivative, the flg22 peptide) and Arabidopsis FLAGELLIN SENSING 2 (FLS2); fungal chitin/chitosan and the Arabidopsis receptor pair CHITIN ELICITOR RECEPTOR KINASE 1 (CERK1)/LYSINE MOTIF RECEPTOR KINASE 5 (LYK5); and NLPs recognized by RECEPTOR-LIKE PROTEIN 23 (RLP23) (Oome et al., 2014; Couto and Zipfel, 2016; Gong et al., 2020). PTI involves a series of defense outputs, ranging from early responses, such as changes in ion fluxes, ROS production, and activation of mitogen-activated protein kinases (MAPK), to late responses, such as callose deposition and growth inhibition (Couto and Zipfel, 2016; Saijo et al., 2018). Avr2 suppresses PTI responses, including ROS accumulation, MAPK activation, callose deposition, and growth inhibition, upon Flg22, chitin, chitosan, or nlp24 application (Di et al., 2017; Tintor et al., 2020; Coleman et al., 2021; de Lamo et al., 2021).

Besides its PTI-suppressing activity, Avr2 also acts as an avirulence factor upon its recognition in the plant nucleus by the resistance protein I-2 (Houterman et al., 2009; Ma et al., 2015) inducing effector-triggered immunity (ETI). Notably, *AVR2* and *SIX5* form a gene pair, and their expression is controlled by a shared intergenic promoter region (Schmidt et al., 2013). *AVR2* and *SIX5* are jointly required not only for full virulence of *Fol* on a susceptible tomato, but also for avirulence on resistant tomato plants expressing *I-2*, highlighting their joint action in plant cells (Ma et al., 2015). Furthermore, Avr2 and Six5 were observed to interact specifically at PD and increase the SEL by an unknown mechanism, permitting translocation of Avr2 to the adjacent cells (Cao et al., 2018).

Here, we study how this effector pair functions together. Thereto, we investigated whether Six5, like Avr2 (Cao et al., 2018), suppresses early and late PTI responses upon PAMP treatment. Furthermore, we monitored the effect on the susceptibility of a host to various plant pathogens using transgenic *SIX5-*expressing Arabidopsis plants. Finally, the SEL aperture and the number of callose deposits of PD in the presence and absence of Avr2 and/or Six5 were determined to unravel the underlying molecular mechanism that promotes cell-to-cell movement of effectors between the plant cells.

## Results

### Δ*spSIX5* Arabidopsis plants show accelerated disease symptoms upon infection with an *AVR2*-expressing *F. oxysporum* strain

To investigate the involvement of Six5 in disease susceptibility, transgenic Arabidopsis (*Arabidopsis thaliana*) lines were generated. Col-0 plants were transformed with a *35S:*Δ*spSIX5* construct, encoding a mature Six5 protein (i.e., without its endogenous signal peptide (Δsp) to ensure cytosolic localization of the effector protein). Two independent homozygous T3 lines, Δ*spSIX5 #2* and Δ*spSIX5 #7*, were selected and used for detailed analysis. The heterologous expression of the *SIX5* transgene was confirmed by RT-PCR (Supplementary Figure 1A), revealing specific PCR products in both transgenic lines that were absent in the wild-type progenitor line. To assess whether *SIX5* expression resulted in readily observable phenotypic changes, both the timing and percentage of seed germination were determined and compared to wild-type Arabidopsis (Supplementary Figure 1B). Δ*spSIX5 #2* and *#7* lines showed similar germination rates as Col-0 (Supplementary Figure 1B). In addition, the morphological phenotype of 4-week-old Δ*spSIX5* (*#2, #7*) Arabidopsis plants was similar to that of wild-type Col-0 (Supplementary Figure 1C).

**Figure 1:**
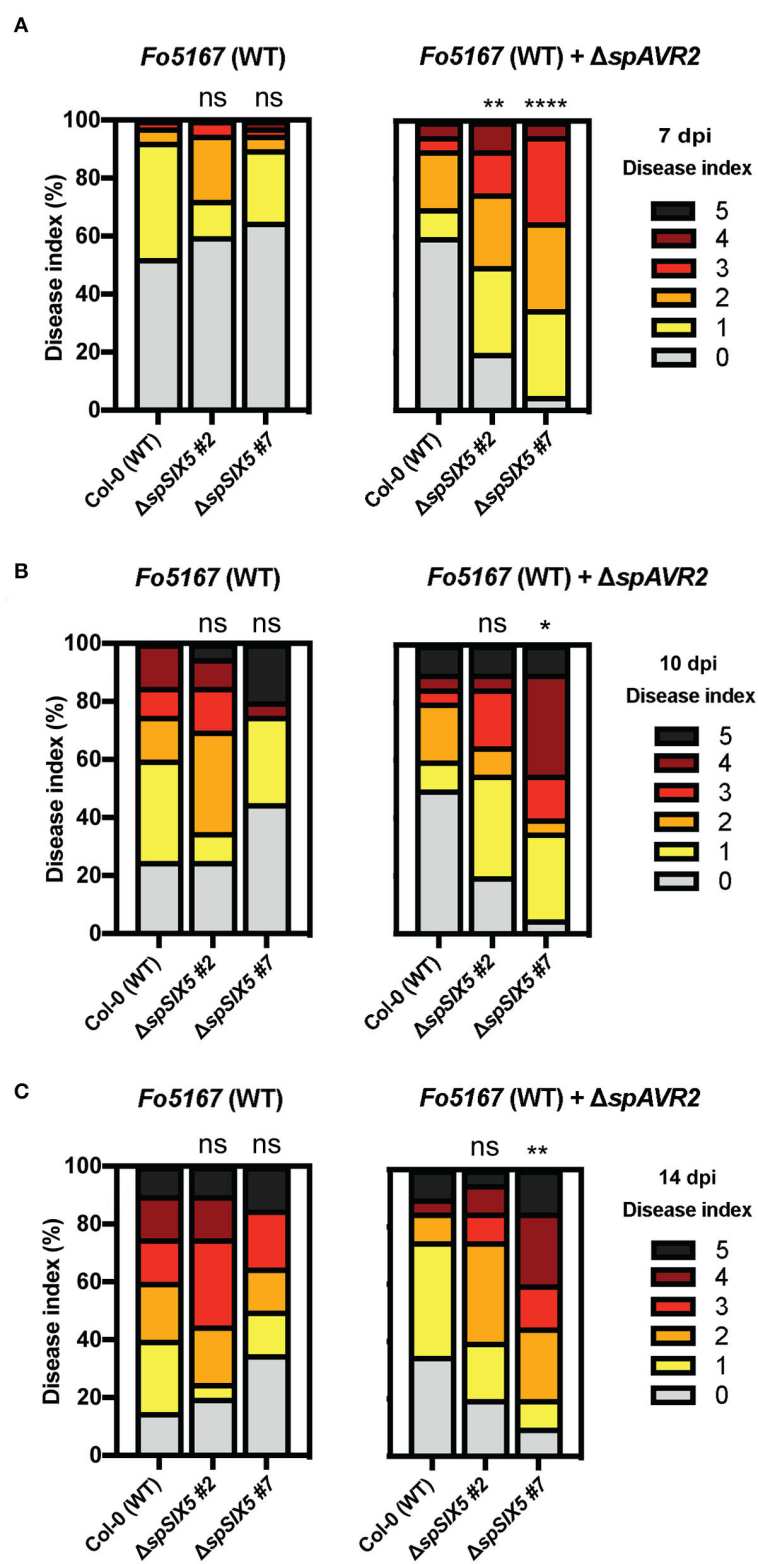
*SIX5-*expressing Arabidopsis plants show increased disease symptom development during the early stages of infection with an *AVR2-*expressing *Fo*5167 strain. Fourteen-day-old Arabidopsis plants (Col-0, Δ*spSIX5 #2*, and *#7*) inoculated with wild-type *Fo*5167 and *AVR2-*expressing transgenic *Fo*5167. Disease index scored on an ordinal scale of 0 (no symptoms) to 5 (plant death) at **(A)** 7 days post-inoculation (dpi), **(B)** 10 dpi, and **(C)** 14 dpi. Each replicate consists of 20 plants per line per treatment, and each time point was repeated 2–4 times (****=*p* < 0.0001, **=*p* < 0.006, *=*p* < 0.02, one-way ANOVA; Kruskal–Wallis, Dunn's multiple comparison test). Of note, **(A)** shows the data of two merged replicates, while **(B,C)** depict a single replicate of the respective time points.

To test whether *SIX5* expression affected the disease susceptibility of Arabidopsis, disease assays were performed using the Arabidopsis-infecting *F. oxysporum* strain *Fo*5167 (Thatcher et al., 2009). Since Six5 has been reported to function in conjunction with Avr2 (Cao et al., 2018), a transgenic *AVR2*-expressing *Fo*5167 was included in the assays (Figure 1 and Supplementary Figure 2). Fourteen-day-old wild-type Col-0 and Δ*spSIX5* seedlings were infected with Fusarium spores by root-dip inoculation and subsequently scored for disease symptom development (Gawehns et al., 2014). The wild-type Col-0 control inoculated with *Fo*5167 showed the first disease symptoms at 7 dpi, which became more severe at later time points (10 and 14 dpi). Of note, no differences in disease development were observed between Δ*spSIX5 #2, #*7, and Col-0 when infected with wild-type *Fo*5167 at any time point (Figures 1A–C). Notably, when infected with *AVR2-*expressing transgenic *Fo*5167, we observed accelerated disease development in Δ*spSIX5 #2* and *#7* plants when compared to Col-0 at 7 dpi, (Figure 1A). At later time points (10 and 14 dpi), the difference in disease symptom development became less pronounced (Figures 1B,C). Taken together, while the expression of *SIX5* alone did not affect the disease susceptibility of Arabidopsis toward *Fo*5176, it did increase disease susceptibility during the early stages of Fusarium infection in the presence of Avr2.

### *SIX5* expression does not change susceptibility to other pathogens or PAMP-triggered immune responses

**Figure 2:**
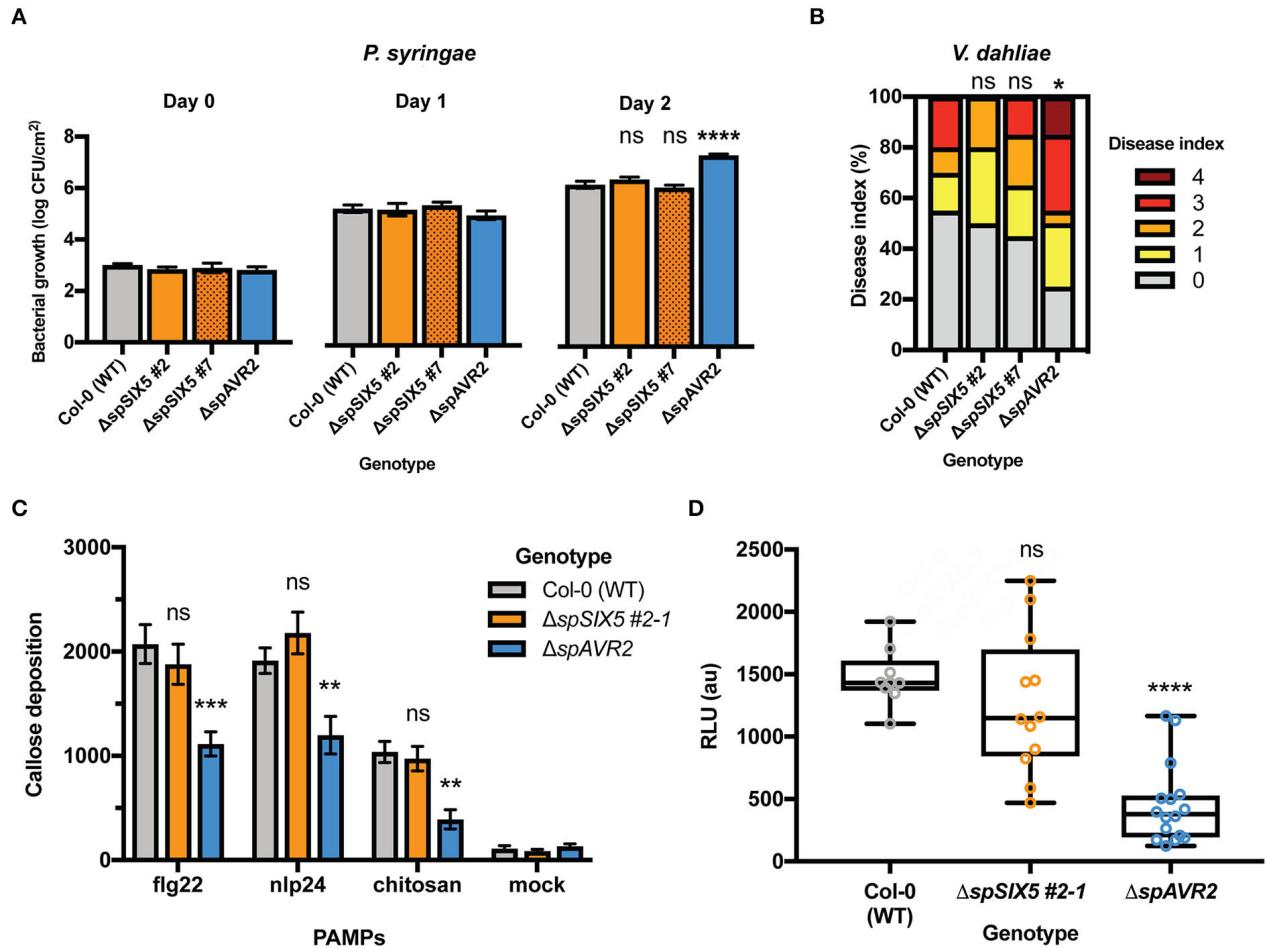
*SIX5* expression does not affect the plant immune response independent of Avr2. **(A,B)**. *SIX5* expression does not affect the susceptibility of Arabidopsis to *P. syringae* and *V. dahliae*. **(A)** *Pseudomonas syringae pv. tomato* DC3000 was infiltrated into 5-week-old leaves of Col-0, Δ*spSIX5 #2, #7*, or Δ*spAVR2* Arabidopsis. Bacterial titers were measured at 0, 1, and 2 dpi (three leaf disks per replicate, error bars indicate standard error of four plants per line). Three biological replicates were performed and representative data from one experiment is depicted (****=*p* < 0.0001, one-way ANOVA). **(B)** Disease index score at 21 dpi after *V. dahliae* inoculation. The disease index is scored on an ordinal scale of 0 (no symptoms) to 5 (plant death). The result from one representative experiment from three independent biological replicates is shown (*=*p* = 0.09, one-way ANOVA; Kruskal–Wallis, Dunn's multiple comparison test). **(C,D)** PTI defense responses are not suppressed by *SIX5* expression; **(C)** Flg22-, nlp24-, or chitin- triggered callose deposition in wild-type, Δ*spSIX5*, or Δ*spAVR2 A. thaliana* leaves. Callose depositions were stained with aniline blue and visualized using fluorescence microscopy. Two biological replicates were performed and representative data of one experiment is depicted (***=*p* <0.001, **=*p* <0.009, one-way ANOVA; Dunnett's multiple comparison test, *N* = 8 frames). Error bars represent standard error. **(D)** Flg22-induced reactive oxygen species (ROS) production in wild-type, Δ*spSIX5*, or Δ*spAVR2-*expressing *A. thaliana* Col-0 leaves. Cumulative relative light unit (RLU) values measured with a luminol assay are depicted. Two biological replicates were performed, and representative data of one experiment is shown (****=*p* <0.0001, one-way ANOVA; Holm-Sidak's multiple comparisons tests, *N* = 12–16 leaf disks). Boxes extend from the 25th to the 75th percentile, whiskers from lowest to highest values, and the bar indicates the median.

To study whether *SIX5* expression compromises the susceptibility of Arabidopsis to pathogens other than *AVR2-expressing F. oxysporum*, disease assays were performed with the bacterium *Pseudomonas syringae pv. tomato (Pst)* and the fungus *Verticillium dahliae. P. syringae pv. tomato* DC3000 was syringe-infiltrated into the leaves of 5-week-old Col-0, Δ*spSIX5 #2, #7*, and Δ*spAVR2* Arabidopsis plants. Δ*spAVR2* plants served as a positive control, as this effector was previously shown to suppress PTI and confer hyper-susceptibility to various pathogens, including *P. syringae* (Di et al., 2017). Leaf disks were taken from the infiltrated areas at 0, 1, and 2 dpi, and bacterial titers were determined (Figure 2A). Consistent with our previous studies, disease susceptibility to *P. syringae pv. tomato* DC3000 was significantly increased in Δ*spAVR2* plants at 2 dpi when compared to that observed in Col-0 plants. However, no altered disease susceptibility was observed in Δ*spSIX5* plants at any time point (Figure 2A). Susceptibility to Verticillium wilt disease was determined in 14-day-old Arabidopsis seedlings root-dip inoculated with *V. dahliae* spores. The development of disease symptoms (chlorosis) in the rosettes was scored according to the disease index (Gawehns et al., 2014). The Δ*spAVR2* plants were more susceptible to *V. dahliae* than the wild-type plants (Col-0) based on the disease symptoms, which confirmed the validity of the disease assay and the use of Δ*spAVR2* as a positive control for increased susceptibility (Supplementary Figure 3). In contrast, Δ*spSIX5* plants showed no increase in the onset of disease symptoms when compared to the wild-type plants (Col-0) (Figure 2B and Supplementary Figure 3).

Although expression of *SIX5* did not affect disease development or bacterial proliferation in Arabidopsis inoculated with *Fo*5167, *P. syringae pv. Tomato*, or *V. dahliae*, the effector might still compromise PTI defense responses like Avr2 (Di et al., 2017). To investigate whether Six5 is capable of suppressing PTI defense responses, both an early (ROS accumulation) and late defense response (callose deposition) were analyzed upon PAMP treatment. To visualize callose deposits, leaf disks were stained with aniline blue following PAMP treatments. Besides the immunogenic peptide flg22, two other PAMPs were applied, i.e., chitosan and nlp24. Chitosan is a derivative of the fungal cell wall component chitin (Gong et al., 2020; de Lamo et al., 2021), while nlp24 is an immunogenic peptide of cytotoxic NLPs that is shared between pathogenic bacteria, fungi, and oomycetes (Oome et al., 2014). Twenty-four hours after flg22, nlp24, chitosan, or mock treatment, leaf disks of Col-0, Δ*spSIX5*, and Δ*spAVR2* plants were collected, and the number of callose deposits was imaged using a stereomicroscope. As expected, mock-treated leaves showed a very low number of callose depositions in Col-0, Δ*spSIX5*, and Δ*spAVR2* (Figure 2C and Supplementary Figure 4), indicating no defense response was triggered. Col-0 showed an increased number of callose spots in response to flg22, nlp24, and chitosan when compared to the mock treatment. Δ*spAvr2* leaves showed a clearly reduced callose deposition in response to all three tested PAMPs, in line with the defense-suppressing activity of Avr2. In the Δ*spSIX5* leaves, the number of callose depositions in response to flg22, nlp24, or chitosan treatment was similar to that of wild-type plants, indicating that Six5 does not have a late-PTI-suppressing activity (Figure 2C and Supplementary Figure 4).

To test whether Six5 can suppress an early PTI defense response independent of Avr2, the impact of Six5 on the ROS burst was assessed. The ROS burst was measured with a luminol-chemiluminescence assay following flg22 treatment of leaf disks of Col-0, Δ*spSIX5*, and Δ*spAvr2* Arabidopsis plants (Figure 2D). The Col-0 control showed a cumulative ROS burst of ~1,400 relative light units (RLU) over a 45-min period following flg22 application. As reported previously, Δ*spAVR2* plants showed a strongly reduced flg22-triggered ROS burst as compared to Col-0 (Di et al., 2017). Of note, Δ*spSIX5* plants showed a wild-type-like ROS accumulation following flg22 treatment, indicating that besides a late PTI output, early PTI signaling was also not affected by Six5 (Figure 2D). Taken together, in contrast to Avr2, Six5 apparently does not interfere with FLS2-, RLP23-, and CERK1-triggered immune responses, nor does it promote disease susceptibility to *P. syringae* or *V. dahliae* in Arabidopsis.

### The Avr2/Six5 effector pair increases the size exclusion limit of plasmodesmata

The Avr2-dependent increase in susceptibility to Fusarium in Δ*spSIX5* Arabidopsis lines implies a role of Six5 during early infection stages. Since Avr2 and Six5 function as an effector pair in manipulating PD and allowing cell-to-cell movement of Avr2 (Cao et al., 2018), we wanted to further investigate the effect of Avr2/Six5 on PD permeability. Therefore, we set out to determine the effect of Avr2/Six5 on the SEL of PD, and its consequence on the ability to translocate other *F. oxysporum* effector proteins.

**Figure 3:**
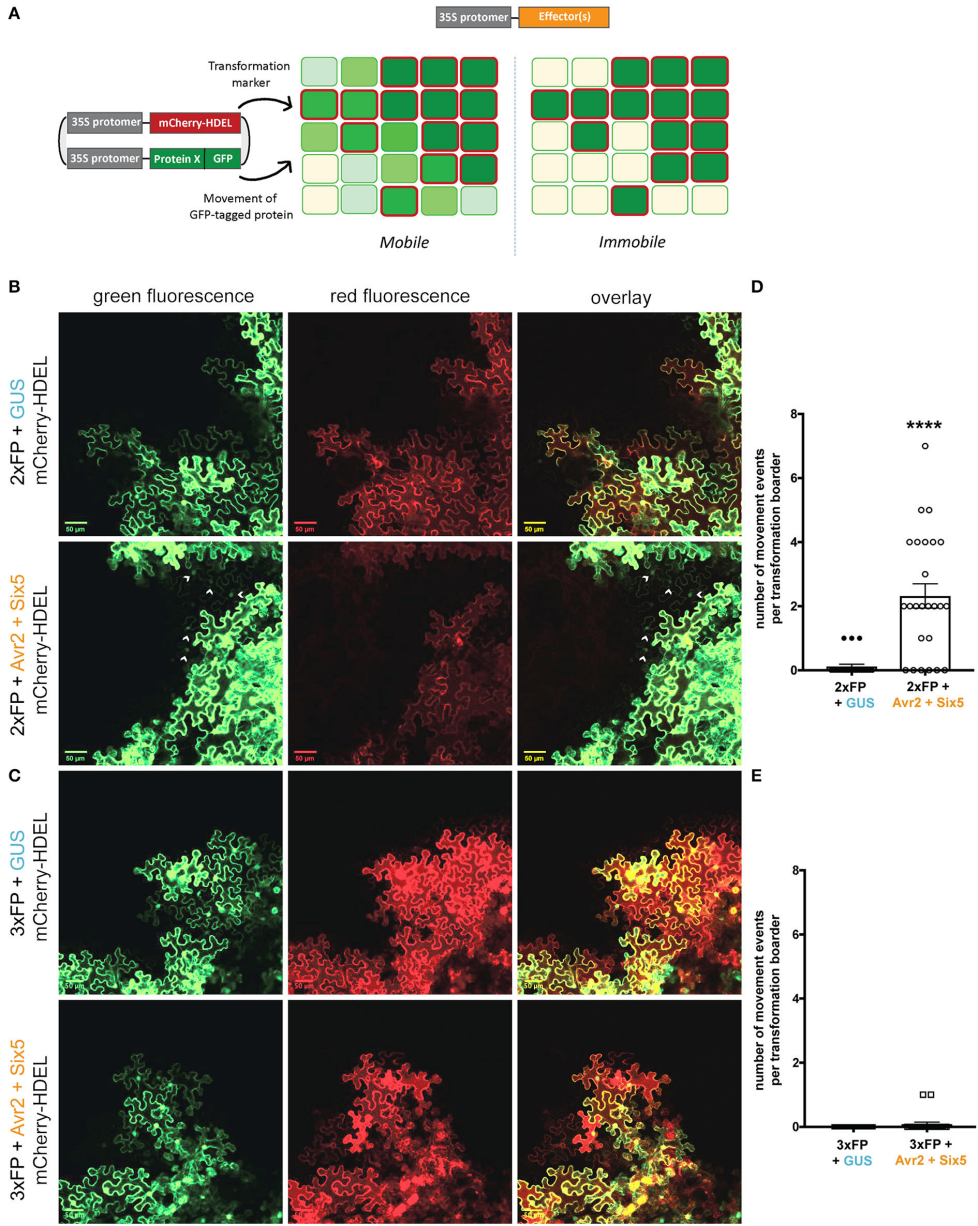
The Avr2/Six5 effector pair increases the size exclusion limit of plasmodesmata in *N*. *benthamiana*. **(A)** Schematic overview of the setup used to visualize the cell-to-cell movement of green fluorescent proteins (GFP) upon agroinfiltration. The binary vector contains two genes, one encoding a GFP-tagged protein fusion and the other an ER-localized mCherry-HDEL protein that functions as a marker for the transformation of individual cells. Two possible localization patterns are depicted; mobile, where the movement of GFP-tagged proteins is represented as fading shades of green, and immobile, where the GFP signal is retained in the primary transformed cells, outlined in red. **(B,C)** Co-infiltration of *A. tumefaciens* strains carrying a binary vector encoding mCherry-HDEL and either 2xFP or 3xNeonGreen (NG) together with a strains expressing either *35S:*Δ*spAVR2/ 35S:*Δ*spSIX5* or *35S:GUS* into *N*. *benthamiana* leaves. Green and red fluorescence was analyzed 72 h after infiltration using confocal microscopy. Transformed cells show both a red ER-localized signal and a green nucleo-cytoplasmic-localized 2xFP or 3xFP signal (indicated by arrowheads) Scale bar represents 50 μm. **(D,E)** Approximately 20–25 individual images based on two independent biological replicates were analyzed to quantify the cell-to-cell movement of 2xFP and 3xFP, respectively. Each data point represents a single image of a transformation border where the number of cell-to-cell movement events (i.e., number of cells) was scored (****=*p* < 0.0001, Mann–Whitney test). Error bars represent the standard error of the mean.

The extent to which the SEL of PD can be altered by Avr2/Six5 manipulation was examined by assessing the mobility of fluorescent fusion proteins of increasing sizes. The setup used to visualize the cell-to-cell movement of green fluorescent proteins is based on a binary vector that encodes both a GFP-tagged protein fusion and an ER-localized mCherry protein. The latter protein carries a C-terminal HDEL ER retention motif that retains mCherry inside the transformed cells, thereby serving as a marker for transformed cells (Figure 3A). Depending on the mobility of the GFP-tagged protein, two possible localization patterns can emerge upon agroinfiltration in *Nicotiana benthamiana*. When the GFP-tagged protein is mobile and can translocate to neighboring cells, this will result in green fluorescent cells surrounding red-labeled transformed cells (Figure 3A, left panel). In the case of an immobile protein, the GFP signal will be retained in the transformed cells and only cells showing both red and green signals will be observed (Figure 3A, right panel). While a single GFP protein (~27 kDa) can typically move freely from cell to cell through PD, a 2xFP [GFP-NeonGreen (NG)] fusion protein (~53 kDa) is severely restricted, as it apparently exceeds the SEL of PD (Kim and Zambryski, 2005; Aung et al., 2020). Since a 2xFP fusion protein is similar in size to Avr2-GFP (~43 kDa) (Supplementary Figure 5A), which can translocate from cell to cell in the presence of Six5 (Cao et al., 2018), we suspected movement of 2xFP in the presence of Avr2/Six5. To determine whether Avr2/Six5-mediated manipulation of PDs allows translocation of larger cargo, the mobility of a 3xFP (NG-GFP-NG, ~80 kDa) (Supplementary Figure 5A) fusion protein was determined. Thereto, *A. tumefaciens* strains carrying a binary vector encoding mCherry-HDEL and either 2xFP or 3xFP were co-infiltrated into *N. benthamiana* leaves with strain(s) expressing *35S:*Δ*spAVR2* and *35S:*Δ*spSIX5* (Figure 3). As a negative control, *35S:GUS* was used instead of *35S:*Δ*spAVR2* and *35S:*Δ*spSIX5*. As expected, in the absence of Avr2/Six5, no green signal was observed in untransformed cells adjacent to a sector of transformed cells both for 2xFP (Supplementary Figure 3B, top panels) and 3xFP (Figure 3C, top panels). In the presence of Avr2/Six5, however, untransformed cells bordering *2xFP* expressing areas showed green fluorescence in their cytoplasm and/or nucleus (marked by arrowheads), indicating cell-to-cell movement of 2xFP (Figure 3B, bottom panels). However, translocation of 3xFP was not observed irrespective of the presence of Avr2/Six5 (Figure 3C, bottom panels). To provide quantification of the cell-to-cell movement patterns for 2xFP and 3xFP proteins, the number of cell-to-cell movement events (i.e., number of GFP-positive cells) adjacent to the sector of transformed cells was counted (Figures 3D,E). In the presence of Avr2/Six5, the frequency of cells showing the cell-to-cell movement of 2xFP increased by 19-fold times (Figure 3D), whereas movement of 3xFP fusion was not observed in any of the samples examined. Taken together, these data show that Avr2/Six5 induces an SEL alteration in *N. benthamiana* epidermal cells, allowing the translocation of a 53 kDa protein, but not of an 80 kDa protein.

### The Avr2/Six5 pair facilitates movement of other Fusarium effector proteins without affecting PD-localized callose

**Figure 4:**
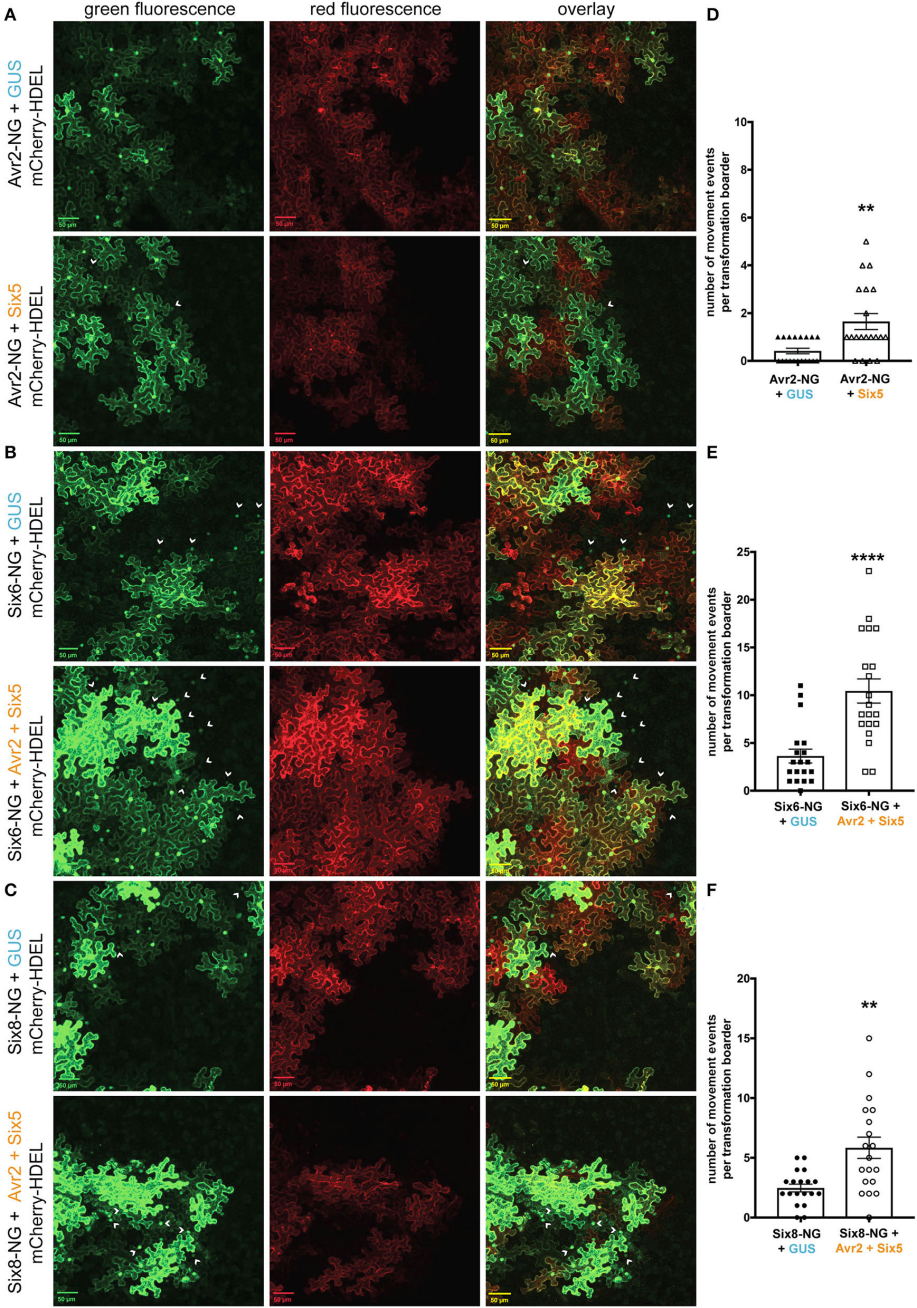
The Avr2/Six5 effector pair facilitate the movement of *Fusarium* effectors proteins, Six6 and Six8. **(A–C)** Co-infiltration of *A. tumefaciens* strains carrying a binary vector encoding mCherry-HDEL and either Avr2-NG, Six6-NG, or Six8-NG together with a strains expressing either *35S*:Δ*spAVR2*/*35S:*Δ*spSIX5* or *35S:GUS* into *N*. *benthamiana* leaves. Fluorescence was analyzed 72 h post-infiltration using confocal microscopy. Transformed cells show both a red ER-localized signal and a green nucleo-cytoplasmic-localized Avr2-NG, Six6-NG, or Six8-NG signal (indicated by arrowheads). The scale bar represents 50 μM. **(D–F)** Approximately, 20 individual images based on two independent biological replicates were analyzed to provide quantification of cell-to-cell movement of, respectively, Avr2-NG, Six6-NG, and Six8-NG. Each data point represents a single image of a transformation border where the number of cell-to-cell movement events (i.e., number of cells) was scored (****=*p* < 0.0001, **=*p* < 0.003, Mann–Whitney test). Error bars represent the standard error of the mean.

*Fusarium oxysporum* effectors other than Avr2 might benefit from an increased SEL of PD to move from cell to cell. To study this, we performed cell-to-cell movement assays using two *F. oxysporum* effectors, Six6 and Six8, that localize and/or function intracellularly (Gawehns et al., 2014; Tintor et al., 2020). Since the Six6-NG and Six8-NG fusion proteins have either a similar or smaller molecular weight than the 2xFP fusion protein (Supplementary Figure 5B), we hypothesized that both effectors would show cell-to-cell mobility in the presence of Avr2/Six5. The Six5-mediated movement of Avr2 served as a positive control for effector translocation in this experiment. Co-agroinfiltration was done using strains carrying a binary vector encoding mCherry-HDEL and either Avr2-NG, Six6-NG, or Six8-NG together with a strain(s) encoding either *35S:*Δ*spAVR2* and/or *35S:*Δ*spSIX5* or *35S:GUS* into *N*. *benthamiana* leaves. The latter construct served again as a negative control for PD alteration by Avr2/Six5 and was used to balance the *A. tumefaciens* concentration in co-infiltrations. In the absence of Avr2/Six5, no green fluorescence signal was observed in the untransformed cells adjacent to Avr2-NG expressing sectors (Figure 4A, top panels). Unexpectedly, Avr2/Six5 independent cell-to-cell movement of Six6-NG and Six8-NG was observed as green fluorescence was observed in the cytoplasm and/or nucleus of neighboring cells (marked by arrowheads) (Figures 4B,C, top panels). Of note, in the presence of Avr2/Six5, an increased number of non-transformed green fluorescent cells flanking transformed cells were observed (Figures 4A–C, bottom panels). Quantifying the mobility events of Avr2-NG, Six6-NG, and Six8-NG in the presence and absence of Avr2/Six5 revealed a 2- to−4-fold increase in the mobility of these effectors in the presence of Avr2/Six5 (Figures 4D–F). Hence, even though Six6-NG and Six8-NG are capable of autonomously translocating to neighboring cells more easily than Avr2-NG, their overall increase in mobility facilitated by Avr2/Six5 is similar.

**Figure 5:**
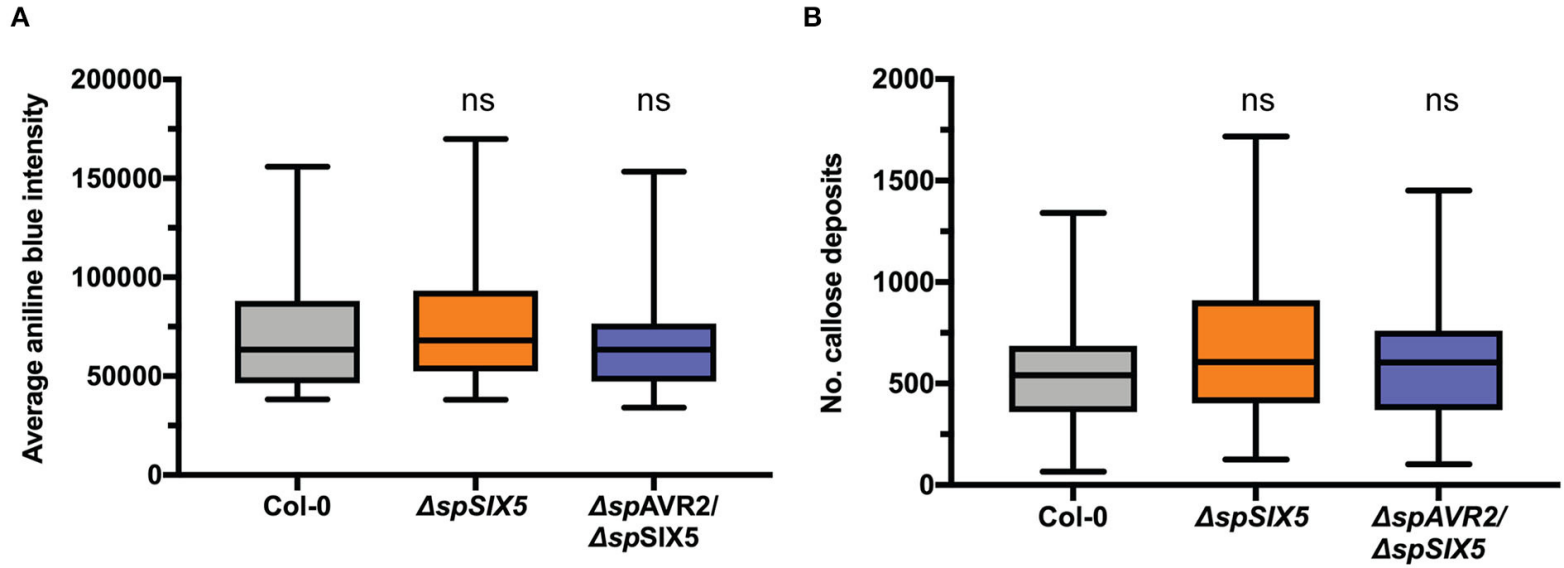
The effector pair Avr2/Six5 does not affect callose deposition at plasmodesmata. **(A)** PD-localized callose depositions in wild-type, Δ*spSIX5*, or Δ*spAVR2/*Δ*spSIX5 A. thaliana* Col-0 leaves. Callose depositions were stained with aniline blue and visualized using confocal microscopy, determining the integrated density of callose deposits per z-stack. About 7–8 biological replicates were performed, of which the average is depicted (one-way ANOVA; Dunnett's multiple comparison test). **(B)** Number of PD detected per z-stack. About 7-8 biological replicates were performed, of which the average is depicted (one-way ANOVA; Dunnett's multiple comparison test, ns: *p* > 0.05). Boxes extend from the 25th to the 75th percentile, whiskers from lowest to highest values, and the bar indicates the median.

Callose deposition at PD controls their aperture. Viruses, bacteria, fungi, and oomycetes commonly target callose turnover to promote virulence (Liu et al., 2021). To study whether Avr2/Six5 targets this mechanism to promote cell-to-cell movement of *Fo* effectors, the amount of callose at PD and the number of PD were determined inΔ*spSIX5* and Δ*spAVR2/*Δ*spSIX5* expressing Arabidopsis plants. No differences were observed in the size or number of callose depositions at PD when compared to that of wild-type Col-0 plants (Figures 5A,B and Supplementary Figure 6). This indicates that the mechanism employed by Avr2/Six5 to manipulate PD aperture does not involve targeting callose homeostasis at these structures or increasing their number.

## Discussion

Here, we show that Six5 apparently acts exclusively to promote the transport and action of other Fusarium effectors. In contrast to Avr2, constitutive expression of *SIX5* in Arabidopsis did not increase disease susceptibility to various pathogens (*F. oxysporum*, V. dahliae, and *P. syringae*) nor did it suppress FLS2-, RLP23-, and CERK1-triggered immune signaling (Di et al., 2017). However, in the presence of Six5, the virulence of a transgenic *AVR2-*expressing Fo5176 strain was increased during the early stages of infection. The Six5-facilitated cell-to-cell movement of Avr2, and potentially other Fo5167 effectors, is apparently particularly important during the early stages of infection. We hypothesize that the endogenous function of Six5 is to aid the spread of Avr2 in root tissues surrounding the infection zone, allowing the effector to suppress PTI ahead of the fungus, thereby contributing to infection. Indeed, *AVR2* transgenic Arabidopsis plants are hyper-susceptible to *Fo*, as in these plants the effector is already present in all cells (Di et al., 2017) (Figure 2). Concomitantly, no accelerated disease symptoms in *AVR2/SIX5-*expressing Arabidopsis plants when compared to *AVR2* lines were observed (unpublished data). The effect of Six5 on the virulence of an *AVR2*-expressing *Fo* strain was less pronounced at the later stages of infection, implying that Avr2 function is particularly important during the colonization of the root endodermis and cortex, but not once the fungus has entered and colonized the vasculature. Given the increasing amount of PD in the root epidermis and cortex cells toward the vasculature, it is not surprising that effectors aimed at promoting translocation between the cells have the strongest effect early in the infection process (Burch-Smith et al., 2011). Taken together, these data show that Six5 itself is not directly involved in manipulating the host to facilitate disease susceptibility, but likely suppresses PTI indirectly by promoting the spread of Avr2 and/or other effectors.

The manipulation of PD by Avr2/Six5 not only promoted the movement of Avr2, but also enhanced the mobility of the Six6 and Six8 effectors. In contrast to Avr2-NG, both Six6-NG and Six8-NG were readily observed in the cells neighboring the transformed cells in the absence of Avr2/Six5. Given the dependency of our cell-to-cell movement assay on the visual detection of the fluorescent signal of effector-NG chimeric proteins, it cannot be excluded that all three effectors to some extent exert the propensity to translocate to neighboring cells. Nonetheless, a similar fold increase in cell-to-cell movement of Avr2, Six6, and Six8 was found in the presence of Avr2/Six5. This implies that besides these effectors, other *Fo* effector proteins, with similar molecular weights, will likely benefit from the enhanced SEL of PD conferred by Avr2/Six5. An interesting question that remains to be answered is whether Six5 co-translocates with Avr2. Unfortunately, our efforts to visualize the direct movement of GFP- or NG-tagged Six5 were unsuccessful due to its relatively low accumulation and hence weak fluorescence (Cao et al., 2018). However, indirect evidence of Six5 mobility can be inferred from the occasional observation of the movement of 2xFP or Six6/Six8-NG across multiple cell layers (e.g., Figure 4B, bottom panels, arrowhead top right corner).

The mechanisms underlying cell-to-cell movement through PD manipulation are diverse and have been mostly studied with plant viruses (Benitez-Alfonso et al., 2010; Schoelz et al., 2011; Reagan and Burch-Smith, 2020). Viral movement proteins (MPs) use various mechanisms of PD manipulation (Benitez-Alfonso et al., 2010; Schoelz et al., 2011; Reagan and Burch-Smith, 2020). Some viral MPs achieve SEL expansion through, e.g., removal of callose deposition by β-glucanase recruitment or prevent callose formation by interference with callose synthases. Although effector mobility and PD alteration by pathogenic microbes is an emerging research topic, the mechanism of how microbial effector proteins manipulate PD is poorly understood (Liu et al., 2021). The RxLR3 effector from *P. brassicae* interacts with and inhibits at least three plant callose synthases (CalS1, CalS2, and CalS3), thereby reducing callose deposition around PD and resulting in an increased SEL (Tomczynska et al., 2020). The *P. syringae* effector HopO1-1 localizes at PD and interacts with PDLPs (plasmodesmata-localized proteins) (Aung et al., 2020). HopO1-1 destabilizes PDLP5 and PDLP7, which are important regulators of callose homeostasis at PD. Several other Hop effector proteins were also shown to be mobile and translocate through PD, depending on their molecular weight (Li et al., 2021). However, regardless of the presence of the mobile *P. syringae* effectors, no increased susceptibility was observed in *SIX5-*expressing Arabidopsis plants. Given our finding that the SEL of PD is not massively increased by Avr2/Six5 allowing passage of proteins of at least 53 kDa but not larger than 80 kDa, a structurally invasive (e.g., tubule formation by viral MPs) PD manipulation mechanism seems unlikely. The observation that (a) the amount of callose at PD and (b) the number of PD is unaffected in Avr2/Six5 plants indicates that the observed increased SELs and effector cell-to-cell mobility is not due to the manipulation of callose homeostasis or the number of PDs by the effector pair. This implies that these effectors might employ a novel mechanism by which they alter PD permeability.

Since Six5 alone cannot facilitate cell-to-cell movement (Cao et al., 2018), the presence of Avr2 and Six5 appears to be necessary for a tripartite interaction at the PD. It is unknown whether Six5 localizes at PD in the absence of Avr2 or *vice versa*, and the strong fluorescence signal of the cytosolic-localized fraction near the cell periphery masks that of a potential PD-localized pool. Pull-down assays using plant material expressing both *Avr2* and *Six5* could identify the effector target and provide insight into the mechanism underlying PD manipulation by these fungal effectors. However, identification of plant targets of the Avr2/Six5 effector pair has proven to be difficult. Pull-down methods using Avr2 and Six5 as baits did not identify candidates, nor did yeast-to-hybrid screens using tomato cDNA libraries as bait (Ma et al., 2015). Future experiments could focus on potential targets located in PD complexes, which would require prior isolation of PD structures (Maule et al., 2011; Salmon and Bayer, 2013). Taken together, our data show that plasmodesmata manipulation by *Fol* requires the combined action of a pair of effector proteins. While the Avr2 effector also has an additional function in suppressing PTI signaling induced by various PAMPs, the major function of Six5 appears to be to act together with Avr2 to facilitate cell-to-cell movement of Avr2 and maybe of other effectors. The unique and overlapping functions of this well-characterized effector pair provide an ideal starting point to investigate the underlying cell biological processes and how they contribute to fungal virulence. Furthermore, it will be interesting to explore whether a similar bifurcation of effector function can be found in other plant-infecting microbes.

## Materials and methods

### Plant material and fungal and bacterial strains

*Arabidopsis thaliana* ecotype Columbia (Col-0), previously described Δ*spAvr2* line (Di et al., 2017) and two Δ*spSIX5* lines (Cao et al., 2018) were used. Arabidopsis seedlings were grown under short day conditions (13/11 h, dark: light cycles at 22°C and 70% humidity). The pathogenic strains of *Fo*5176 (Thatcher et al., 2012), *Avr2-*expressing *Fo*5176 (Tintor et al., 2020), *Verticillium dahliae* race 1 JR2 (Fradin et al., 2009), and *Pseudomonas syringae pv. tomato* (*Pst*) DC3000 (Whalen et al., 1991) have been described previously.

### RNA isolation and reverse-transcription polymerase chain reaction (RT-PCR) to verify SIX5 *expression in Arabidopsis*

Total RNA was extracted using TRIzol LS reagent (Invitrogen, Waltham, MA, USA) from leaf material (400 mg of rosette leaves) of 14-day-old Arabidopsis seedlings ground in liquid nitrogen. DNA was removed by on-column RNase-free DNase (Qiagen, Hilden, Germany) treatment. cDNA was synthesized from 1 μg of total RNA using M-MulV reverse-transcriptase RNaseH minus kit with oligo dT primers (Fermentas, Waltham, MA, USA). Arabidopsis actin primers FP3147 and FP3148 (Supplementary Table 1 primer list) were used as an internal control for the normalization of the RT-PCR. Primer pair FP872 and FP873 was used to amplify Six5 from cDNA.

### Vector construction

Binary vectors (pZK538 backbone) to allow the monitoring of cell-to-cell movement of 2xFP, 3xFP, Six6-NG, Six8-NG, or Avr2-NG, respectively, were generated using a previously described protocol (Blekemolen et al., 2018). The following modifications were made, pDONR207: Neongreen (Shaner et al., 2013; Botman et al., 2019) was used to amplify the coding sequence of Neongreen, with primer set FP8002 and FP8003. To replace GFP in the pZK538 backbone with Neongreen, the pZK538 vector was linearized using inversion PCR excluding the GFP sequence, with primer set FP8283 and FP8284. The obtained linearized pZK538 (minus GFP) was ligated with the amplified Neongreen fragment using the Hifi DNA assembly cloning protocol (NEB), to generate pZK538-Neongreen. The 2xFP-pZK538 (NG-GFP) was generated by ligating the Neongreen fragment together with linearized pZK537-GFP [primer set previously described (Blekemolen et al., 2018)]. 3xFP-pZK538 (NG-GFP-NG) was generated by amplifying the coding sequence of the Neongreen-GFP fusion (Primer set FP8292 and FP 8293) and ligating it together with linearized pZK539-Neongreen (primer set FP8287 and FP7939). Avr2-NG, Six6-NG, and Six8-NG pZK538 vectors were generated by amplifying Δ*spAvr2* (Houterman et al., 2009), Δ*spSix6* (Gawehns et al., 2014), and Δ*spSix8* (Gawehns et al., 2014) from pDONR207: Δ*spAvr2*, pDONR207: Δ*spSix6*, and pDONR207: Δ*spSix8*, respectively (primer sets *Avr2* FP8288; FP8289, *Six6* FP10596; FP10697, *Six8* FP8458; FP8459), and recombining the fragment together with linearized pZK538-Neongreen.

### Agrobacterium tumefaciens-mediated transient transformation of *N. benthamiana* leaves

Agroinfiltration of 5-week-old *N. benthamiana* leaves was performed as described by a previous method (Ma et al., 2012). Co-infiltration of *A. tumefaciens* GV3101 strains, *2xFP/mCherry-HDEL, /mCherry-HDEL, Avr2-NG/mCherry-HDEL, Six6-NG/mCherry-HDEL*, and *Six8-NG/mCherry-HDEL* together *3xFP* with either *35S:*Δ*spAvr2, 35S:*Δ*spSix5*, or *35S:GUS*, were carried out to obtain an OD_600_ of 0.5 in each sample. The viral silencing suppressor p19 was co-infiltrated along with the other constructs at an OD_600_ of 0.1. The leaves were subjected to confocal microscopy 48 h after infiltration.

### Confocal microscopy

Confocal microscopy was performed with a Nikon A1 (Nikon Instruments Inc., Melville, NY, USA). GFP and Neongreen fluorophores were excited at 488 nm with an Ar-ion laser, and the signal emitted was selected using a 500–525/550 nm bandpass filter. Excitation of mCherry was carried out at 561 nm with a diode-pumped solid-state (dpss) laser, and the light signal emitted was selected with a 570/595–620 nm bandpass filter. Multi-labeling with mCherry and GFP or Neongreen was imaged by sequential scanning to monitor the co-localization of the proteins.

### *F. oxysporum, V. dahliae*, and *P. syringae* infection assays of Arabidopsis

Inoculation of Arabidopsis with *F. oxysporum* and *V. dahliae* was performed by dipping the roots in a fungal spore suspension (10^6^ spores/mL) as described previously (Gawehns et al., 2014; Di et al., 2017). After repotting, the seedlings were placed in a growth chamber with a 13/11 dark/light regime at 28°C, and the disease symptoms were scored after 1–3 weeks (Supplementary Figures 2, 3). *Pst* DC3000 inoculation was done by syringe-infiltration of a bacterial suspension (OD_600_ of 0.0005) into the leaves of 4-week-old *Arabidopsis thaliana* plants as described previously (Di et al., 2017). The bacterial titers (as colony-forming units) were determined by a serial dilution of leaf disk samples taken at 0–2 dpi.

### Reactive oxygen species (ROS) assay

The ROS production was measured using a luminol/peroxidase-based assay (Felix et al., 1999). Of note, leaf disks (5 mm in diameter) of 4-week-old Col-0, Δ*spSIX5 #2*, and Δ*spAvr2 A. thaliana* plants were collected and floated (adaxial side up) overnight in deionized water (MQ) water in a Petri dish. Subsequently, leaf disks were transferred to a 96-well plate (Perkin Elmer, Waltham, MA, USA) containing 100 μl of water per well. Immediately before measurement, 100 μl of a luminol/peroxidase solution was added to each well. Final concentrations were 250 μM luminol (Sigma, Saint Louis, MO, USA), 10 μg/ml peroxidase (Sigma Saint Louis, MO, USA), and 100 nM flg22 (Genscript HK limited, Hong Kong). Luminescence was recorded at 3-min intervals for 45 min using a plate reader (Synergy H1; BioTek, Winooski, VT, USA).

### Callose staining

To visualize callose spots, 3–4 week-old *A. thaliana* leaves were syringe-infiltrated with either flg22 (100 nM), nlp24 (1 μM, Genscript HK limited, Hong Kong), chitosan (100 μg/ml), or water. Chitosan with a low molecular weight (50–190 kDa, 75–85% deacetylated, Sigma-Aldrich, Saint Louis, MO, USA) was prepared as described previously (Rendina et al., 2019). The prepared chitosan stock was diluted to a concentration of 5 mg/ml. Twenty-four hours after infiltration, the leaves were collected, de-stained, and preserved in 70% ethanol: acetic acid (3:1). Cleared *A. thaliana* leaves were washed and rehydrated in 50% v/v ethanol. Subsequently, the leaves were stained for 60–120 min with a 0.01% aniline blue solution (product no. 21999.183, VWR Chemicals, Radnor, PA, USA) (pH of 9, dissolved in 0.07 M sodium phosphate buffer) and mounted in 50% glycerol. For callose visualization of the *A. thaliana* leaves, a Leica MZ FLIII fluorescence microscope (Wetzlar, Germany) equipped with a DAPI filter (UV filter, excitation 360 nm, emission 420 nm) was used. The number of callose depositions per image (magnification of ×5) was counted using a script in ImageJ.

Callose deposition at PD was visualized as described previously (Xu et al., 2017). Of note, the eighth rosette leaf of 5-week-old plants was infiltrated with aniline blue (0.01% in PBS buffer, pH 7.4). Callose deposits were imaged from the abaxial side of the leaf using an SP5 confocal microscope (Leica) with excitation at 405 nm and emission collected between 440 and 470 nm, using a 63× oil immersion lens (Plan-APOCHROMAT 63×/1.4 oil). 3–10 z-stacks were collected from seven or eight independent plants. Callose was quantified using automated image analysis defining plasmodesmata within the 5–50 voxel range (https://github.com/JIC-CSB/find-plasmodesmata).

### Protein isolation and western blot

Total protein fraction was isolated from *N. benthamiana* by grinding 3–4 leaf disks (5 mm in diameter) in liquid N_2_ and resuspending the ground tissue in 200–300 μL of extraction buffer (50 mM Tris-HCL (pH 7.5), 1% v/v NP-40, 5 mM DTT, and 1× protease inhibitor cocktail; Roche, Basel, Switzerland), and centrifuging at 4°C at 15,000 g for 30 min. Proteins (10 μL of each sample) were separated using SDS-PAGE (Bio-rad system, Hercules, CA, USA) using 10% gels. Immunoblotting was carried out using the semidry blotting method (Thermoscientific Owl HEP-1 system, Waltham, MA, USA) on PVDF (polyvinylidenedifluoride) membranes. Blots were blocked in TBST (0.1% tween) with 5% v/v milk and probed with rat or mouse monoclonal GFP- or NG-antibodies (Chromotek, Planegg-Martinsried, Germany) at a dilution of 1:3,000 or 1:500. Secondary polyclonal antibodies goat anti-rat or -mouse antibody (Pierce, Waltham, MA, USA) were used at a dilution of 1:5,000. The signal was visualized with the ECL kit (GE Healthcare, ECL prime of ThermoScientific, Super Signal West Pico, Chicago, IL, USA), according to the manufacturer's instructions and detected using a Chemidoc imaging system (Bio-rad, Hercules, CA, USA).

## Data availability statement

The original contributions presented in the study are included in the article/Supplementary material, further inquiries can be directed to the corresponding authors.

## Author contributions

MB, LC, NT, TG, and DP conducted the experiments. MB, NT, TG, DP, CF, and FT designed the experiments. MB and FT wrote the paper. All authors contributed to the article and approved the submitted version.

## Funding

FT, MB, NT, and TG were supported by the NWO-Earth and Life Sciences funded VICI project (no. 865.14.003). CF and DP were supported by the European Research Council (grant 725459), INTERCELLAR and Biotechnology and Biological Research Council Institute Strategic Programme Plant Health BBS/E/J/000PR9796.

## Conflict of interest

The authors declare that the research was conducted in the absence of any commercial or financial relationships that could be construed as a potential conflict of interest.

## Publisher's note

All claims expressed in this article are solely those of the authors and do not necessarily represent those of their affiliated organizations, or those of the publisher, the editors and the reviewers. Any product that may be evaluated in this article, or claim that may be made by its manufacturer, is not guaranteed or endorsed by the publisher.

## Supplementary material

Supplementary Figure 1 Heterologous expression of *Six5* in Arabidopsis does not affect seed germination or plant growth. (A). Ethidium bromide-stained agarose gel showing reverse-transcriptase PCR (RT-PCR) products synthesized on mRNA isolated from leaves of 10-day-old Col-0 and two independent homozygous *35S:*Δ*spSIX5 (#2, #7*) Arabidopsis lines. Expression of housekeeping gene *ACTIN* serves as a positive control for cDNA synthesis. (B). Germination *of 35S:* Δ*spSIX5 #2, #7*, and Col-0 (WT) Arabidopsis seedlings is depicted 1 and 2 days after imbibition of seeds on wet filter paper. Both the timing and percentage of overall seed germination were comparable between Col-0, and *35S* Δ*spSIX5* #2 and #7. (C). Growth of 4-week-old Col-0 (WT) and *35S:*Δ*spSIX5* (*#2, #7*) transgenic Arabidopsis lines under short day conditions does not reveal any apparent phenotypic changes in, e.g., plant size, leaf morphology, or leaf color. Representative images are shown. The scale bar represents 1 cm.

Supplementary Figure 2 Disease index of *Fo*5176 infection in Arabidopsis. The disease index of *Fo*5176 infection in Arabidopsis is scored on an ordinal scale of 0 (no symptoms), 1 (stunting or 1-2 leaves with yellow veins, 2 (fully developed leaves show chlorosis or yellow veins), 3 (most fully developed leaves show chlorosis), 4 (all fully developed leaves show chlorosis), to 5 (plant death).

Supplementary Figure 3 *SIX5* expression does not affect the susceptibility of Arabidopsis to *V. dahlia***.** Col-0, Δ*spSIX5 #2, #7*, and Δ*spAVR2 A. thaliana* plants 21 days post-mock or *V. dahliae JR2* inoculation. Fourteen-day-old Arabidopsis plants were root-dip inoculated with either water or *V. dahliae* spore suspension. Images were taken 21 days post-inoculation.

Supplementary Figure 4 Callose deposition triggered by flg22, nlp24, and chitosan not repressed in Δ*spSIX5* Arabidopsis plants. Flg22-, nlp24-, or chitin- triggered callose deposition in wild-type, Δ*spSIX5*, or Δ*spAVR2 A. thaliana* leaves. Callose depositions were stained with aniline blue and visualized using fluorescence microscopy.

Supplementary Figure 5 Protein accumulation and size determination of GFP fusion and effector fusion proteins by Western blot. (A,B) Immunoblots showing accumulation of GFP (fusion) proteins (1xGFP, 2xFP (NG-GFP), and 3xFP (NG-GFP-NG)) expressed in *N. benthamiana* by agroinfiltration. Molecular weight (kDa) markers are indicated on the left. Equal protein loading is verified by Ponceau S staining of the blots. (A) An α-GFP antibody is used. (B). An α-Neongreen antibody is used. Of note, the same blot was first probed with α-NG and later re-probed with α-GFP to visualize 1xGFP as the size reference. (C). Immunoblot showing accumulation of effector fusion proteins (Avr2-NG, Six6-NG, and Six8-NG) expressed in *N. benthamiana* by agroinfiltration. Molecular weight (kDa) markers are indicated on the left. Equal loading is verified by Coomassie brilliant blue (CBB) staining of the blot.

Supplementary Figure 6 Callose deposition at plasmodesmata is not affected in **Δ*spSIX5* and** Δ***spSIX5/***Δ***spAVR2*** **Arabidopsis plants** Maximum projections of z-stack images showing the amount of callose at plasmodesmata and the number of callose depositions of Col-0,Δ*spSIX5*, or Δ*spSIX5*/Δ*spAVR2 A. thaliana* leaves visualized with aniline blue staining and confocal microscopy.
