# Supplementary figures and images for: The primary function of Six5 of Fusarium oxysporum is to facilitate Avr2 activity by together manipulating the size exclusion limit of plasmodesmata
Source: Front Plant Sci. 2022 Jul 29;13:910594. doi: 10.3389/fpls.2022.910594 (PMC9373983; doi:10.3389/fpls.2022.910594)

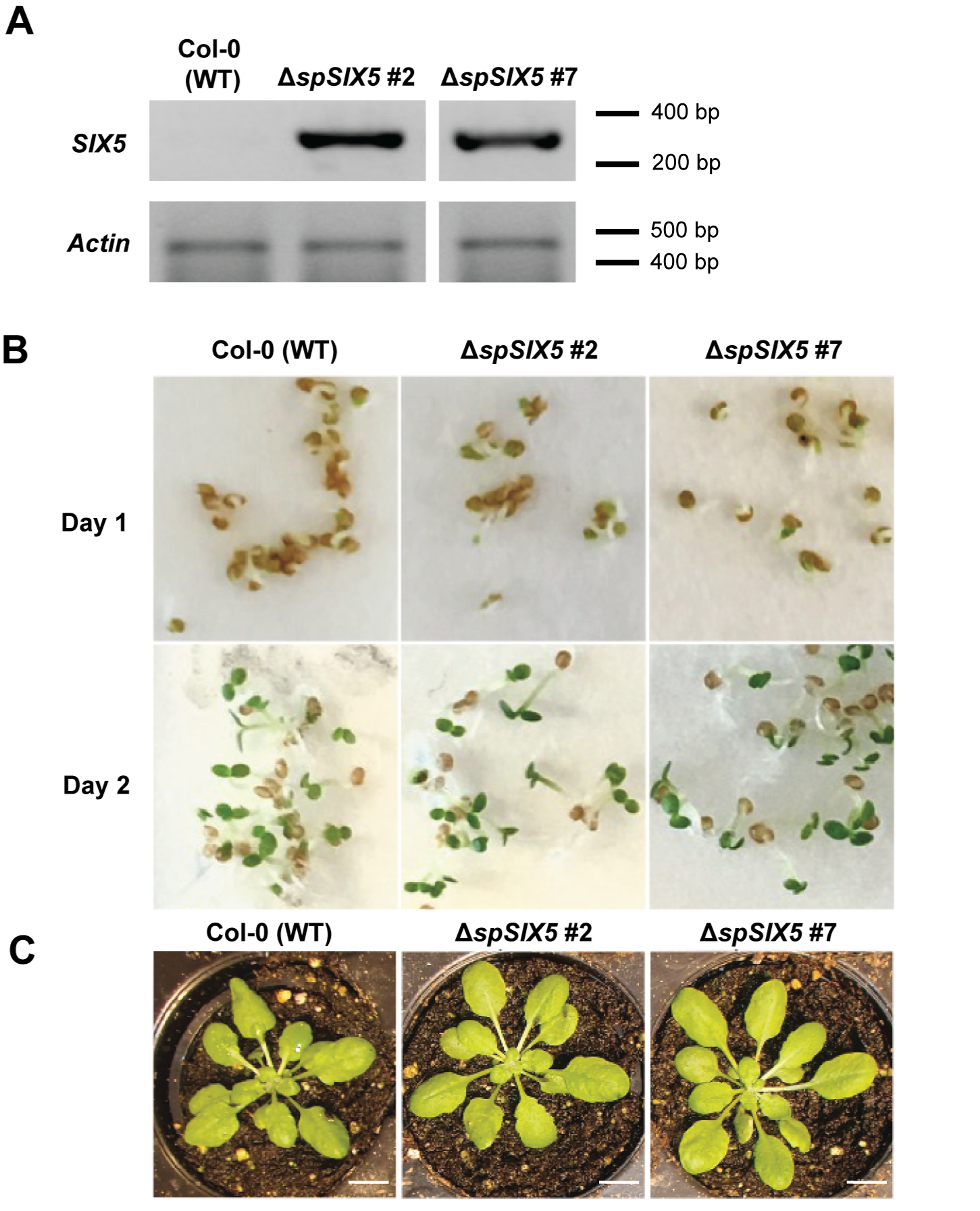

Supplement: Supplementary Figure 1 — Heterologous expression of Six5 in Arabidopsis does not affect seed germination or plant growth. (A). Ethidium bromide-stained agarose gel showing reverse-transcriptase PCR (RT-PCR) products synthesized on mRNA isolated from leaves of 10-day-old Col-0 and two independent homozygous 35S:ΔspSIX5 (#2, #7) Arabidopsis lines. Expression of housekeeping gene ACTIN serves as a positive control for cDNA synthesis. (B). Germination of 35S: ΔspSIX5 #2, #7, and Col-0 (WT) Arabidopsis seedlings is depicted 1 and 2 days after imbibition of seeds on wet filter paper. Both the timing and percentage of overall seed germination were comparable between Col-0, and 35S ΔspSIX5 #2 and #7. (C). Growth of 4-week-old Col-0 (WT) and 35S:ΔspSIX5 (#2, #7) transgenic Arabidopsis lines under short day conditions does not reveal any apparent phenotypic changes in, e.g., plant size, leaf morphology, or leaf color. Representative images are shown. The scale bar represents 1 cm. [file Image_1.TIF]

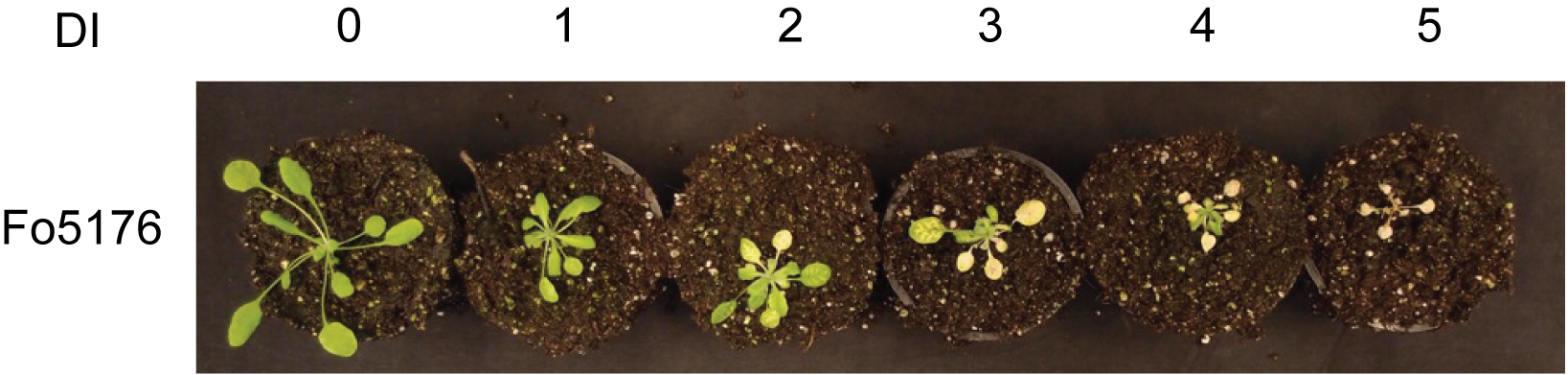

Supplement: Supplementary Figure 2 — Disease index of Fo5176 infection in Arabidopsis. The disease index of Fo5176 infection in Arabidopsis is scored on an ordinal scale of 0 (no symptoms), 1 (stunting or 1-2 leaves with yellow veins, 2 (fully developed leaves show chlorosis or yellow veins), 3 (most fully developed leaves show chlorosis), 4 (all fully developed leaves show chlorosis), to 5 (plant death). [file Image_2.TIF]

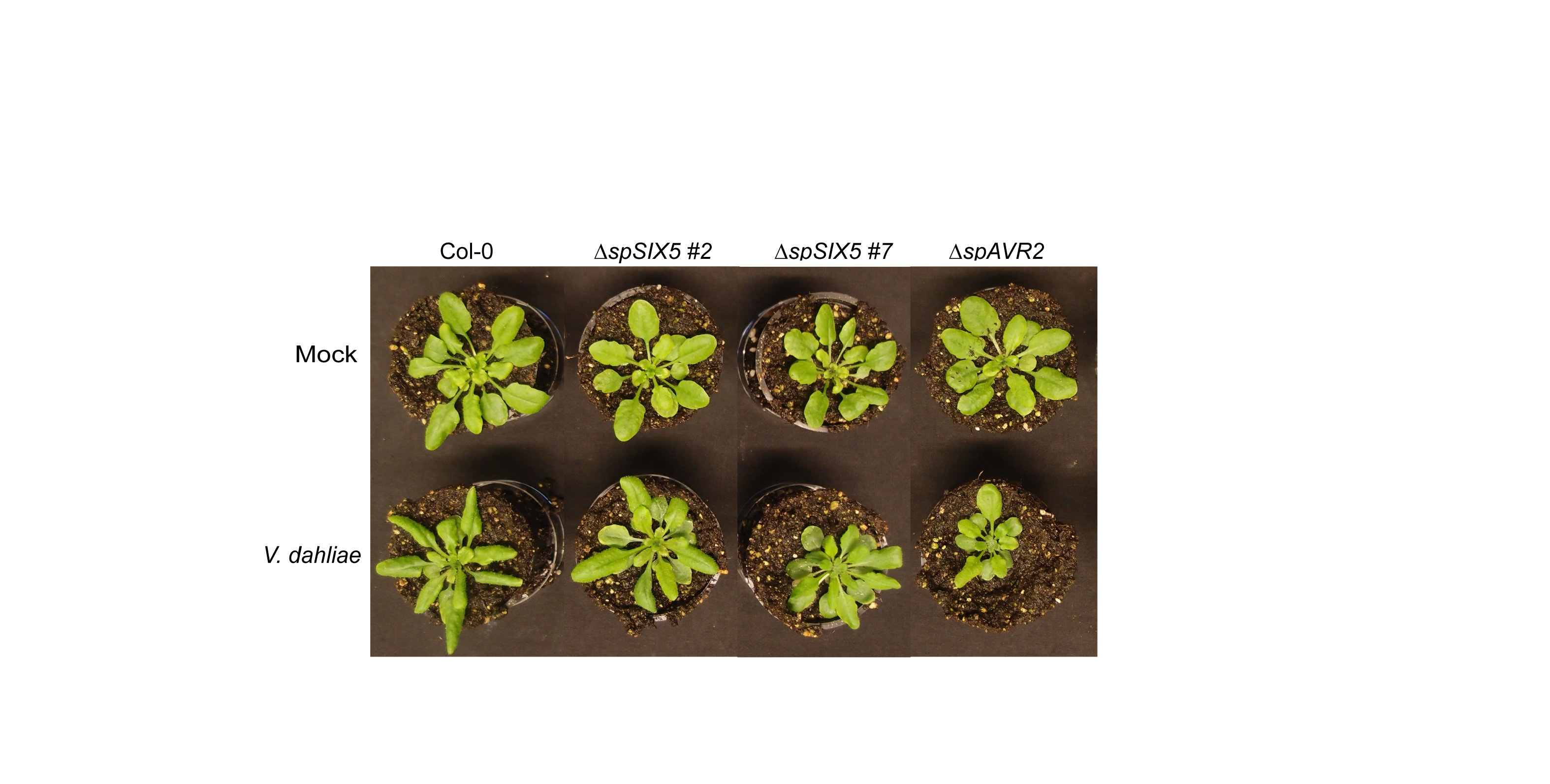

Supplement: Supplementary Figure 3 — SIX5 expression does not affect the susceptibility of Arabidopsis to V. dahlia. Col-0, ΔspSIX5 #2, #7, and ΔspAVR2 A. thaliana plants 21 days post-mock or V. dahliae JR2 inoculation. Fourteen-day-old Arabidopsis plants were root-dip inoculated with either water or V. dahliae spore suspension. Images were taken 21 days post-inoculation. [file Image_3.TIF]

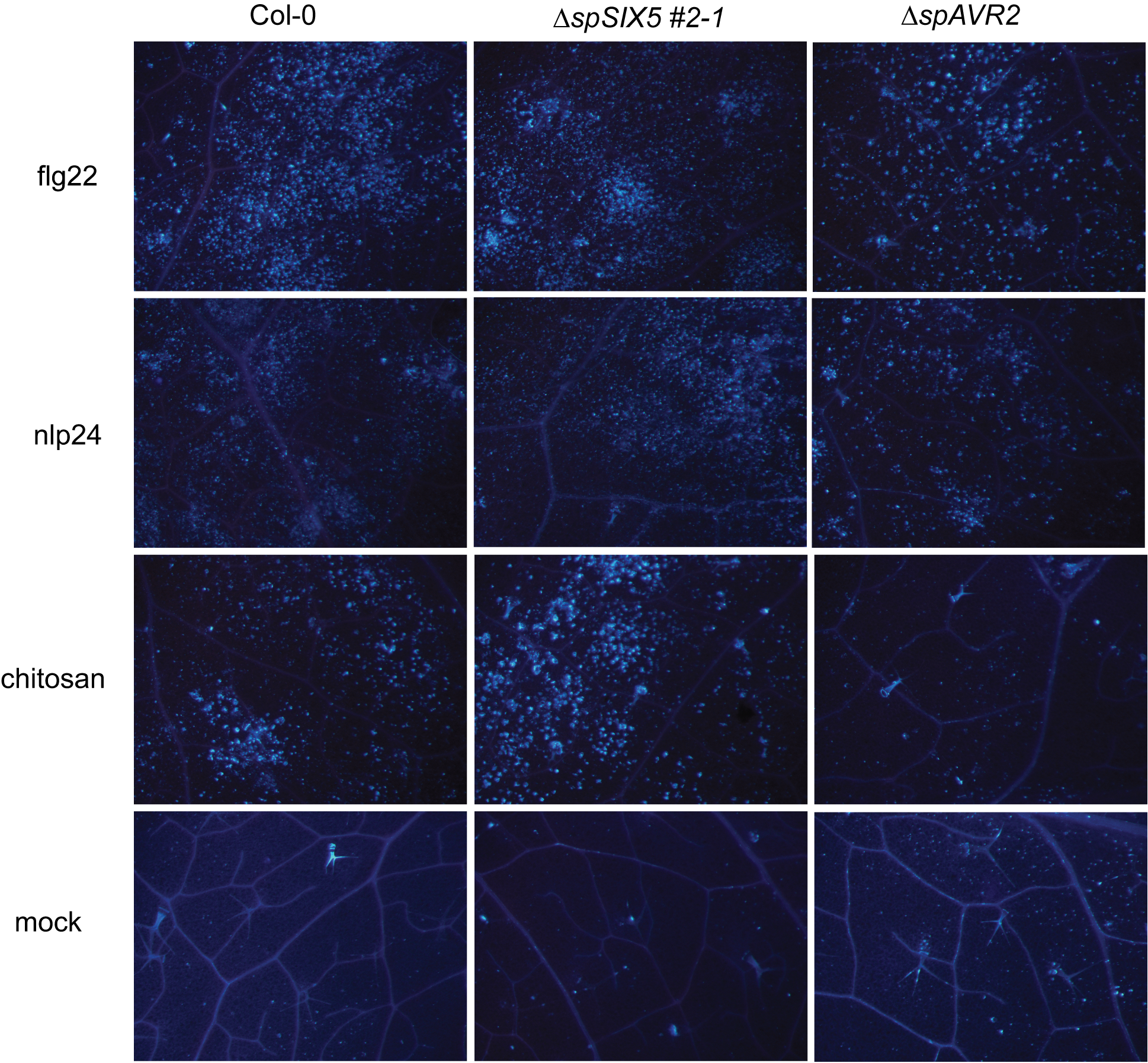

Supplement: Supplementary Figure 4 — Callose deposition triggered by flg22, nlp24, and chitosan not repressed in ΔspSIX5 Arabidopsis plants. Flg22-, nlp24-, or chitin- triggered callose deposition in wild-type, ΔspSIX5, or ΔspAVR2 A. thaliana leaves. Callose depositions were stained with aniline blue and visualized using fluorescence microscopy. [file Image_4.TIF]

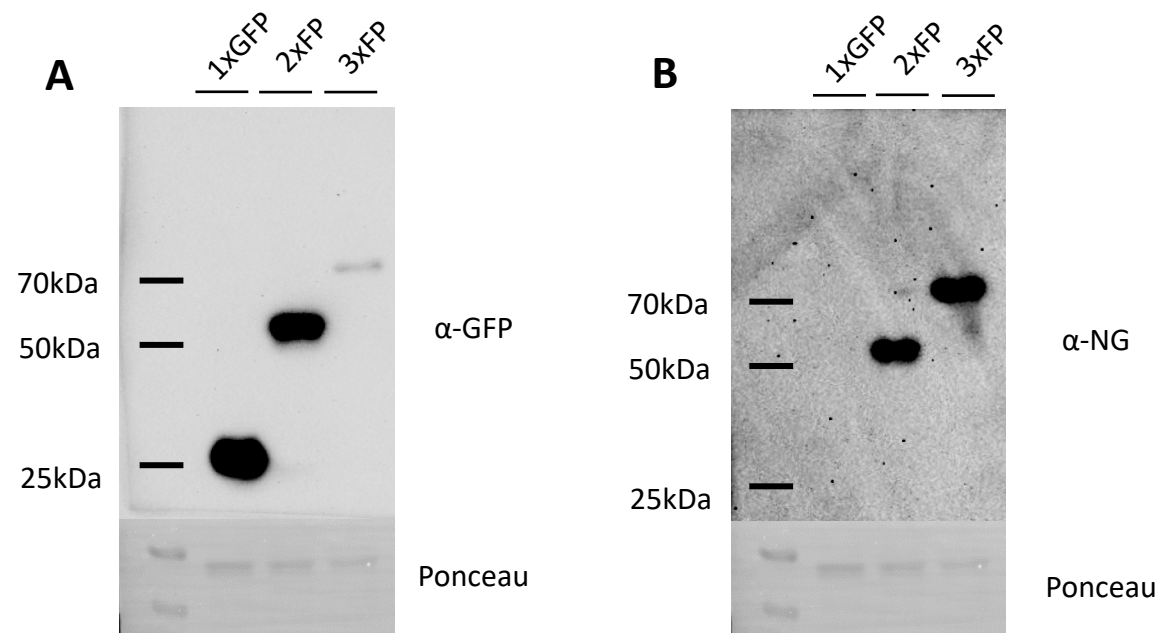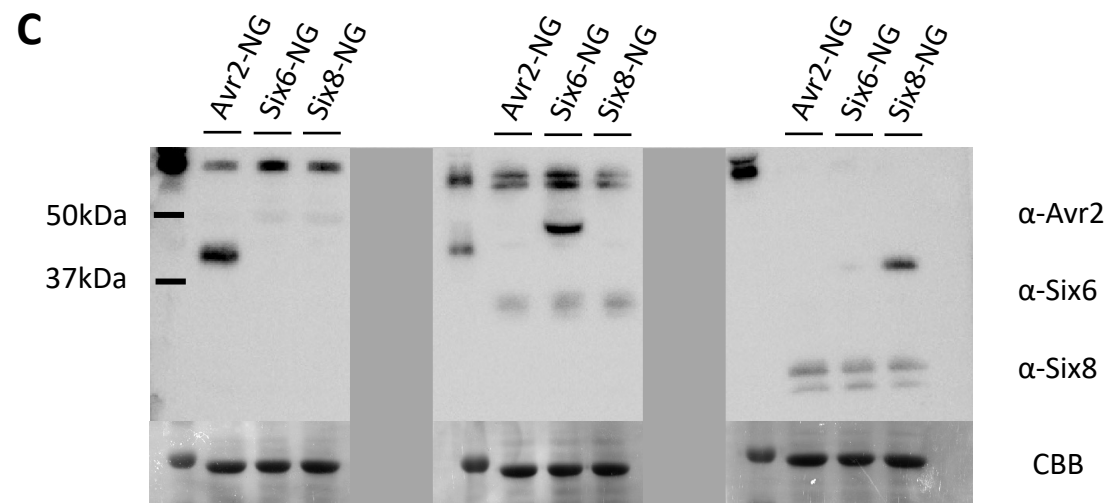

Supplement: Supplementary Figure 5 — Protein accumulation and size determination of GFP fusion and effector fusion proteins by Western blot. (A,B) Immunoblots showing accumulation of GFP (fusion) proteins (1xGFP, 2xFP (NG-GFP), and 3xFP (NG-GFP-NG)) expressed in N. benthamiana by agroinfiltration. Molecular weight (kDa) markers are indicated on the left. Equal protein loading is verified by Ponceau S staining of the blots. (A) An α-GFP antibody is used. (B). An α-Neongreen antibody is used. Of note, the same blot was first probed with α-NG and later re-probed with α-GFP to visualize 1xGFP as the size reference. (C). Immunoblot showing accumulation of effector fusion proteins (Avr2-NG, Six6-NG, and Six8-NG) expressed in N. benthamiana by agroinfiltration. Molecular weight (kDa) markers are indicated on the left. Equal loading is verified by Coomassie brilliant blue (CBB) staining of the blot. [file Data_Sheet_1.pdf]

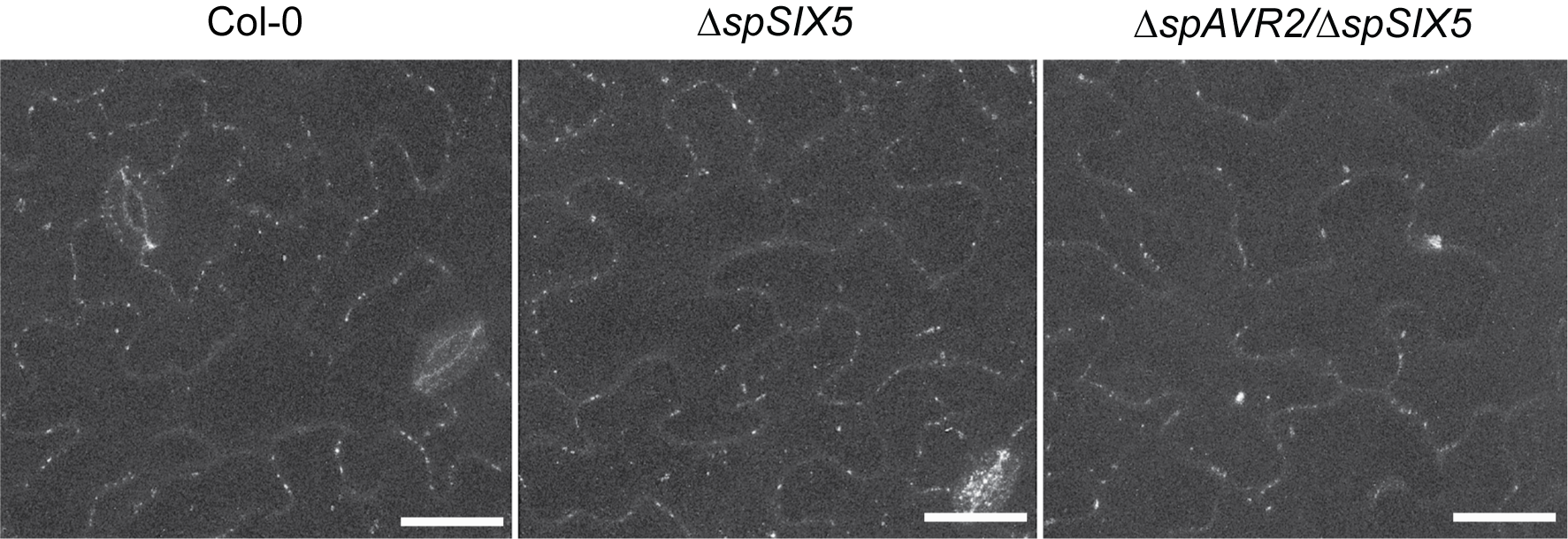

Supplement: Supplementary Figure 6 — Callose deposition at plasmodesmata is not affected in ΔspSIX5 and ΔspSIX5/ΔspAVR2 Arabidopsis plants Maximum projections of z-stack images showing the amount of callose at plasmodesmata and the number of callose depositions of Col-0,ΔspSIX5, or ΔspSIX5/ΔspAVR2 A. thaliana leaves visualized with aniline blue staining and confocal microscopy. [file Image_6.tif]
