# Supplementary material for: The primary function of Six5 of Fusarium oxysporum is to facilitate Avr2 activity by together manipulating the size exclusion limit of plasmodesmata
Source: Front Plant Sci. 2022 Jul 29;13:910594. doi: 10.3389/fpls.2022.910594 (PMC9373983; doi:10.3389/fpls.2022.910594)
Supplement: Supplementary file 7 [file Image_5.pdf]

Primer number	Sequence (5' to 3')	Target (gene)
FP3147	GAGCTGTGTTTCCTAGTATTGTGGG	AtActin
FP3148	CAAGATCAAGACGTAGGATAGCATG	AtActin
FP872	GGGGACAAGTTTGTACAAAAAAAGCAGGCT	Six5
FP873	GGGGACCACTTTGTACAAGAAAGCTGGGT	Six5
FP8002	CACCATTTACGAACGATAGCCATGGCCTCTCTCCCAGCG	Neongreen
FP8003	ACCACCCCGGTGAACAGCTCCTCGCCCTTGCTCACCATGGATCCCATCACATCGGTAAAGGCCTTTTG	Neongreen
FP8283	TTTCTCCATAATAATGTGTGAG	Inversion PCR pZK538
FP8284	CTATATCTCATCAAAAGATAACTGAG	Inversion PCRpZK538
FP8292	AAATTTTCACCATTTACGAACGATAGCCATGGCCTCTCTCCCAGCG	NG-GFP-NG fragment
FP8293	TCGCTGGGAGAGAGGCCATGGATCCCTTGTACAGCTCGTCCATGC	NG-GFP-NG fragment
FP8287	GGATCCATGGCCTCTCTC	Inversion PCR pZK538-neongreen
FP7939	GGCTATCGTTCGTAAATGGTG	Inversion PCR pZK538-neongreen
FP8288	AAATTTTCACCATTTACGAACGATAGCCATGCCTGTGGAAGATGCC	Avr2
FP8289	TCGCTGGGAGAGAGGCCATGGATCCATCCTCTGAGATAGTAAGATAGTAGGTATAAC	Avr2
FP10596	AAATTTTCACCATTTACGAACGATAGCCATGAAGCTCGCTCTTATCGC	Six6
FP10697	TCGCTGGGAGAGAGGCCATGGATCCCTCCCAAGACCAGGTGTAGG	Six6
FP8458	AAATTTTCACCATTTACGAACGATAGCCATGACGCCTATTGATAAGAGCC	Six8
FP8459	TCGCTGGGAGAGAGGCCATGGATCCGCGGAAATTGTGTAGAAACTGG	Six8
